# SHV Lactamase Engineering Database: a reconciliation tool for SHV β-lactamases in public databases

**DOI:** 10.1186/1471-2164-11-563

**Published:** 2010-10-13

**Authors:** Quan K Thai, Juergen Pleiss

**Affiliations:** 1 Institute of Technical Biochemistry, University of Stuttgart, Allmandring 31, 70569 Stuttgart, Germany

## Abstract

**Background:**

SHV β-lactamases confer resistance to a broad range of antibiotics by accumulating mutations. The number of SHV variants is steadily increasing. 117 SHV variants have been assigned in the SHV mutation table (http://www.lahey.org/Studies/). Besides, information about SHV β-lactamases can be found in the rapidly growing NCBI protein database. The SHV β-Lactamase Engineering Database (SHVED) has been developed to collect the SHV β-lactamase sequences from the NCBI protein database and the SHV mutation table. It serves as a tool for the detection and reconciliation of inconsistencies, and for the identification of new SHV variants and amino acid substitutions.

**Description:**

The SHVED contains 200 protein entries with distinct sequences and 20 crystal structures. 83 protein sequences are included in the both the SHV mutation table and the NCBI protein database, while 35 and 82 protein sequences are only in the SHV mutation table and the NCBI protein database, respectively. Of these 82 sequences, 41 originate from microbial sources, and 22 of them are full-length sequences that harbour a mutation profile which has not been classified yet in the SHV mutation table. 27 protein entries from the NCBI protein database were found to have an inconsistency in SHV name identification. These inconsistencies were reconciled using information from the SHV mutation table and stored in the SHVED.

The SHVED is accessible at http://www.LacED.uni-stuttgart.de/classA/SHVED/. It provides sequences, structures, and a multisequence alignment of SHV β-lactamases with the corrected annotation. Amino acid substitutions at each position are also provided. The SHVED is updated monthly and supplies all data for download.

**Conclusions:**

The SHV β-Lactamase Engineering Database (SHVED) contains information about SHV variants with reconciled annotation. It serves as a tool for detection of inconsistencies in the NCBI protein database, helps to identify new mutations resulting in new SHV variants, and thus supports the investigation of sequence-function relationships of SHV β-lactamases.

## Background

Since the application of penicillin to the clinical practice in the 1940s, the effectiveness of β-lactam antibiotics have been reduced drastically [1-3]. One of the main reasons is the hydrolysis of their β-lactam ring by β-lactamases (EC 3.5.2.6) resulting in a loss of function. These enzymes, especially SHV and TEM β-lactamase variants, accumulate mutations gradually [4,5] to resist β-lactam antibiotics and rapidly spread over the world [6-8].

SHV β-lactamases belong to class A β-lactamases and have a serine in the active site [9]. The premature protein consists of 286 amino acids. The first 21 amino acids at the N-terminus form the signal sequence and are removed to yield the mature enzyme [10]. SHV β-lactamases were first described in the members of the genus Klebsiella as a narrow-spectrum β-lactamase against penicillin [6,11]. Their genes are located either in the bacterial chromosome or on a plasmid [12]. Genes encoding these enzymes have been mutated rapidly and transferred to other Gram-negative bacteria in different geographical regions [6]. Currently, 117 SHV variants have been described. A list of assigned SHV variants was compiled and maintained by Jacoby and Bush [13] which is referred further in this paper as "SHV mutation table". Beside the SHV mutation table, sequence information on SHV b-lactamases can also be found in the NCBI protein database [14]. One of the important data sources of the NCBI protein database is the NCBI nucleotide database which is open for submission of new sequences without further validation; therefore it is growing rapidly, but contains inconsistencies. In contrast, the SHV mutation table is manually curated by experts in the b-lactamase field and therefore is widely accepted as a reliable and consistent information source. In the SHV mutation table, each SHV variant is characterized by its name and mutation profile which is a set of amino acid substitutions at certain positions in the sequence. Positions are identified according to the Ambler numbering scheme [15]. To become listed in the SHV mutation table as a new SHV b-lactamase, it must have arisen naturally, is fully sequenced, and harbors a new mutation profile [13]. Therefore, engineered proteins are not considered.

The SHV Engineering Database (SHVED) was built up as a comprehensive inventory by collecting data on SHV b-lactamases from these two databases to facilitate detection of inconsistencies in entries derived from NCBI protein database and to eventually reconcile them, to detect new SHV β-lactamases with novel mutation profiles, and to identify new amino acid positions at which mutations can occur.

## Construction and content

### Construction

#### Development and construction of SHVED

Amino acid sequence of SHV-1 originated from *Klebsiella pneumoniae *(GenInfo (GI): 4337048) was used as a seed sequence for building up the SHVED. A BLAST search [16] was performed against the NCBI protein database [14] without filtering of low complexity regions and with a low E-value threshold (10^-124^) to prevent the occurrence of TEM lactamases and other non-SHV lactamases in the BLAST results. For each hit in the BLAST result, the GI was extracted and the complete XML entry was downloaded from the NCBI protein database. Information on sequence, position-specific annotations, functional descriptions, and source organism was extracted from the entry and parsed by an automated retrieval system into an in-house developed relational database system [17]. For BLAST results representing protein structures, monomers were extracted from the PDB [18] and deposited as structure entries.

Sequences generated from the annotated mutation profiles deposited in the SHV mutation table [13] were also incorporated into the SHVED. Except for 16 assigned SHVs which were "withdrawn" or "not yet released", 117 assigned SHV sequences were generated and parsed into the SHVED using the available information on amino acid exchanges and the reference sequence SHV-1. On the webpage, the "source organism" of these sequences was set to "Clinical sample" and the data source to 'lc' abbreviated from "Lahey Clinic" where the SHV mutation table is hosted.

#### Identification and naming of SHV β-lactamase sequences

Each protein sequence in the SHVED was aligned with SHV-1 using ClustalW [19] to identify its mutation profile. This mutation profile is the set of amino acid exchanges, deletions, and insertions occurring in a certain SHV, e.g. L35Q for the substitution of leucine at position 35 by glutamine. Subsequently, the mutation profile was matched against the mutation profiles listed in the SHV mutation table to identify whether the respective protein sequence is identical to an already assigned SHV. If the mutation profiles were identical, the protein was named accordingly (e.g. "SHV-3"). Otherwise it was named "SHV-like" and its mutation profile was stored. In the case of sequences longer than SHV-1, only the region corresponding to SHV-1 was examined to identify the mutation profile. Amino acid insertions arising inside the protein sequence were annotated, e.g. "-162.1D -162.2R" for the insertion of two residues aspartic acid and arginine after the residue at position 162. The amino acid deletion was annotated with the corresponding residue and position, e.g. "G54-" for the deletion of a glycine at position 54.

For sequences longer than SHV-1, the number of additional residues was recorded, e.g. "C+5" for a sequence 5 residues longer at its C terminus. Sequences shorter than SHV-1 were considered as fragments of the respective SHV sequences or the SHV-like sequences, although they were probably named differently in the entry of the source database. The number of missing residues at the N- and C- terminus were annotated, e.g. "N-21 C-3" for 21 and 3 residues missing at the N- and C- terminus, respectively.

#### Multisequence alignment and feature annotation

The annotation information was enriched by performing multisequence alignment using CLUSTALW [19]. Information on secondary structure calculated using DSSP [20] were also included in the SHVED. Individual residues in the sequence as well as in the alignments were numbered according to the standard scheme suggested by Ambler [15]

#### Reconciliation of data inconsistencies

A systematic comparison of entries of the NCBI protein database and the SHV mutation table allows a reconciliation of NCBI protein database entries which have an inconsistent annotation. In the SHVED, the wrong name assignment is corrected if its mutation profile is already included in SHV mutation table. A sequence with a new mutation profile is stored in the SHVED as new SHV β-lactamase, even if it has been named by the authors by a (wrong) SHV name in the NCBI protein database. A link from the reconciled SHVED entry to the original NCBI protein database entry allows the author of the respective entry to correct an erroneous entry.

### Content

#### Data content of the SHVED

452 protein sequence entries from NCBI protein database and 117 protein sequences from SHV mutation table were collected and parsed into the SHVED, resulting in 200 distinct protein entries. 20 crystal structures of 2 SHV β-lactamases (SHV-1 and SHV-2) were stored in the SHVED. 19 crystal structures were from SHV-1 with one or two engineered mutations. Apart from the structure (PDB entry 3D4F) which is full-length sequence, all crystal structures lack the 21 residues of the N-terminal signal sequence. Two protein sequences (PDB entries 2A3U and 2A49) possess 5 and 4 additional residues, respectively, at their C-terminus (Table 1).

**Table 1:** PDB code of crystal structure entries in SHVED and their sequence annotations

Protein	PDB ID	Description
SHV-1	3D4FA	structure of SHV-1
	
	1ONGA	structure (N-21) of SHV-1
	1Q2PA	
	1SHVA	
	1VM1A	
	2G2UA	
	2H5SA	
	2ZD8A	
	3C4OA	
	3C4PA	
	
	1TDGA	structure (N-21) of SHV-1 with the artificial S130G mutation and tazobactam[S130G]
	1TDLA	
	
	2A3UA	structure (N-21 C+5) of SHV-1 with the artificial E166A mutation and sulbactam[E166A]
	
	2G2WA	structure (N-21) of SHV-1 with the artificial D104K mutation [D104K]
	
	2A49A	structure (N-21 C+4) of SHV-1 with the artificial E166A mutation and clavulanic acid [E166A]
	
	1RCJA	structure (N-21) of SHV-1 with the artificial E166A mutation [E166A]
	
	2H0TA	structure (N-21) of SHV-1 with artificial M69V, E166A mutations [M69V E166A]
	2H0YA	
	2H10A	

SHV-2	1N9BA	structure (N-21) of SHV-2 [G238S]

Of the 200 proteins, 35 SHV sequences were derived from SHV mutation table, but not from the NCBI protein database, 82 protein sequences were exclusively found in the NCBI protein database, and 83 protein sequences were accessible in both source databases. In 82 protein sequences found only in the NCBI protein database, there are 41 sequences which originate from microbial sources and harbor a new mutation profile. 22 are full-length sequences (table 2) and 19 are fragments (table 3).

**Table 2:** New mutation profiles of full length sequences originating from microorganisms

GI	Source Organism	Description *
74058441	*Klebsiella pneumoniae*	SHV-like [A52T]

224223446	*Klebsiella pneumoniae*	SHV-like [E166K]

154269503	*Klebsiella pneumoniae*	SHV-like [K256R]

160948441	*Klebsiella pneumoniae*	SHV-like [N253H]

218091981	*Klebsiella pneumoniae*	SHV-like [S271I]

161367444	*Klebsiella pneumoniae*	SHV-like [R202S]

218091983	*Klebsiella pneumoniae*	SHV-like [D104G]

218091988	*Klebsiella pneumoniae*	SHV-like [Y7F G238S]

262044380	*Klebsiella pneumoniae*	SHV-like [P252L]

41584432	*Escherichia coli*	SHV-like [T235N G238S E240K]

218684515	*Klebsiella pneumoniae*	SHV-like [H289L]

51150101	*Klebsiella pneumoniae*	SHV-like [R6L G238S E240K]
		
51105097	*Escherichia coli*	

30230495	*Acinetobacter baumannii*	SHV-like [L35Q R191H G238S E240K]

33943602	*Acinetobacter baumannii*	SHV-like [S14Y L35Q G238S E240K]

52630976	*Klebsiella pneumoniae*	SHV-like [L35Q G238S E240K I260V]

41584438	*Escherichia coli*	SHV-like [L35Q T235N G238S E240K]

257359515	*Klebsiella pneumoniae*	SHV-like [T18A A22V L35Q M129V]

41584428	*Escherichia coli*	SHV-like [L35Q T235N G238S E240K S271N]

224979335	*Salmonella enterica*	SHV-like [L35Q K94E G238S E240K]

83596180	*Acinetobacter baumannii*	SHV-like [L35Q G238S E240K N254D]

84380855	*Klebsiella pneumoniae*	SHV-like [Y7F L35Q G238S E240R]

15718691	*Klebsiella pneumoniae*	SHV-like [H96T Y97H -162.1D -162.2R -162.3W -162.4E -162.5T]

* SHV-like: SHV variant with a mutation profile that is not in the SHV mutation table. The mutatation profile is given in square brackets.

**Table 3:** Fragments with new mutation profiles

GI	Microbial source	Description *
41584430	*Escherichia coli*	fragment (C-1) of SHV-like [L35Q T235N G238S E240K]

40950646	*Escherichia coli*	fragment (C-2) of SHV-like [L35Q D233E G238S E240K]

164665324	*Escherichia coli*	fragment (C-3) of SHV-like [G238S E240X]

90403947	*Klebsiella pneumoniae*	fragment (C-4) of SHV-like [L35Q G238S E240K E288F]

94502905	*Klebsiella pneumoniae*	fragment (N-1 C-3) of SHV-like [R6E Y7G I8D R9S L35Q H289L]

90403949	*Escherichia coli*	fragment (N-2 C-7) of SHV-like [Y7W I8V R9I L10F C11P L35Q G238S E240K]

90403945	*Klebsiella pneumoniae*	fragment (N-9 C-1) of SHV-like [L35Q G238S E240K E288N H289L W290G Q291T]

78333	*Escherichia coli*	fragment (N-21) of SHV-like [A140T T141A]

90403951	*Klebsiella pneumoniae*	fragment (N-10 C+1) of SHV-like [L35Q G238S E240K A284R A285P L286Y I287K E288N H289L W290E Q291P R292K]

157838542	*Escherichia coli*	fragment (N-19 C-2) of SHV-like [R43S G238S E240K]

46309198	*Klebsiella pneumoniae*	fragment (N-12 C-11) of SHV-like [P252L]

159138973	*Klebsiella pneumoniae*	fragment (N-23 C-5) of SHV-like [L35Q E168A G251S]

159138975	*Klebsiella pneumoniae*	fragment (N-24 C-5) of SHV-like [L35Q E168A G251S]

56463227	*Escherichia coli*	fragment (N-25 C-13) of SHV-like [E89Q G238S E240K]

56463237	*Escherichia coli*	fragment (N-25 C-13) of SHV-like [V80M G238S E240K]

56463225	*Escherichia coli*	fragment (N-25 C-13) of SHV-like [R43S G238S E240K]

56463229	*Escherichia coli*	fragment (N-25 C-13) of SHV-like [R43S E89Q G238S E240K]

56463239	*Escherichia coli*	fragment (N-25 C-13) of SHV-like [V44G V80M E89Q G238S E240K G251V M272R]

56463235	*Escherichia coli*	fragment (N-25 C-13) of SHV-like [R43S D213A T235P G238S E240K E258D V261L]

* SHV-like: SHV variant with a mutation profile that is not in the SHV mutation table

N-x C-y: sequence lacks × amino acids at the N terminus and y amino acids at the C terminus. The mutatation profile is given in square brackets.

#### Analysis of amino acid substitutions and substitution positions

**Figure 1:**
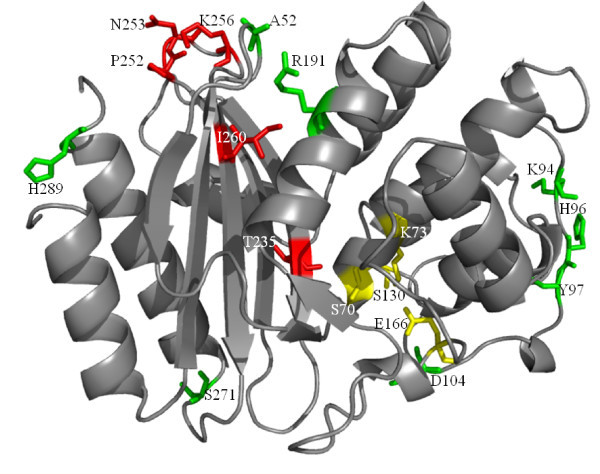
**The structure of SHV-1 β-lactamases (PDB entry 1SHV) with new substitution positions found in full length sequences**. Amino acid side chains are shown in stick representation: substitutions occurring at novel positions (green), novel amino acid substitution at known position (red), active site residues (yellow).

**Figure 2:**
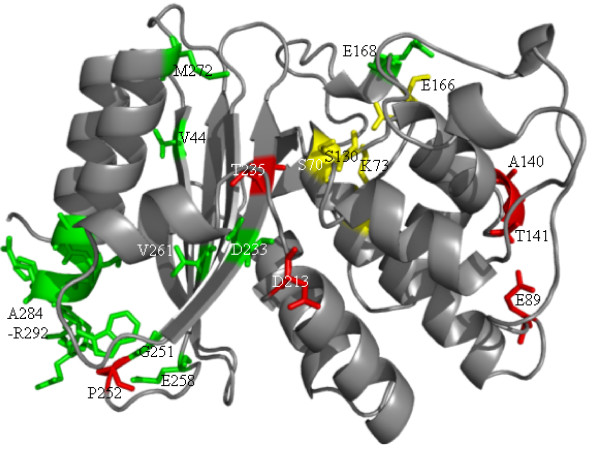
**The structure of SHV-1 β-lactamases (PDB entry 1SHV) with new substitution positions found in fragments**. Amino acid side chains are shown in stick representation: substitutions occurring at novel positions (green), novel amino acid substitution at known position (red), active site residues (yellow).

In addition to the amino acid substitutions described in the SHV mutation table [13], 27 new substitution positions in protein entries originating from microbial sources have been identified. 11 new substitution positions found in full length sequences (table S1, Additional file 1) and 18 new substitution positions were found in fragments (table S2, Additional file 1), in which 2 new substitution positions could be found both in full length sequences and in fragments (positions 6 and 289). These new substitution positions spread over the complete protein sequence, including the signal peptide and the C-terminus. Most of the substitutions found in full length sequences are located at the protein surface and are distant from the active site, except for T235 and I260 (figure 1). Of the 18 new substitution positions found in fragments, 9 positions are at the C terminus, 4 positions on the protein surface, 3 positions in the protein core, and 2 in the signal peptide (figure 2). Not only the substitution at new positions, but also new amino acid exchanges at already known positions were found. As an example, the protein sequence with GI 259038268 harbors an lysine at the position 252 instead of a proline. In the SHV mutation table, only the substitution P252G is described.

#### Data inconsistencies

There are 27 distinct protein entries derived from the NCBI protein database having inconsistent annotations (table 4). In all cases, the annotated SHV name is inconsistent with its mutation profile. For example, the protein sequence with GI 40950644 has three mutations (L35Q, G238S, and E240K), therefore, it should be named "SHV-12" according to the SHV mutation table, but it is actually annotated as "beta-lactamase SHV-5" in the NCBI protein database. In 12 cases, the protein sequence is a fragment and therefore there is not enough information to rename it in the SHVED.

**Table 4:** Inconsistencies between information from NCBI protein database and SHV mutation table

GI	Name and mutation profile according to		Inconsistency
	NCBI protein database	SHV mutation table*	
262044380	SHV-5 [G238S E240K]	SHV-like [P252L]	different mutation profile
	
41584432	SHV-5 [G238S E240K]	SHV-like [T235N G238S E240K]	
	
51105097	SHV-5 [G238S E240K]	SHV-like [R6L G238S E240K]	
	
40950644	SHV-5 [G238S E240K]	SHV-12 [L35Q G238S E240K]	
	
41584438	SHV-5 [G238S E240K]	SHV-like [L35Q T235N G238S E240K]	
	
41584428	SHV-5 [G238S E240K]	SHV-like [L35Q T235N G238S E240K S271N]	

41584434	SHV-5 [G238S E240K]	fragment (C-1) of SHV-12 [L35Q G238S E240K]	a fragment with different mutation profile
	
41584430	SHV-5 [G238S E240K]	fragment (C-1) of SHV-like [L35Q T235N G238S E240K]	
	
40950646	SHV-5 [G238S E240K]	fragment (C-2) of SHV-like [L35Q D233E G238S E240K]	
	
157838542	SHV-7 [I8F R43S G238S E240K]	fragment (N-19 C-2) of SHV-like [R43S G238S E240K]	

77702548	SHV-11 [L35Q]	SHV-1 (no mutation)	different mutation profile

90403947	SHV-12 [L35Q G238S E240K]	fragment (C-4) of SHV-like [L35Q G238S E240K E288F]	a fragment with different mutation profile
	
90403949	SHV-12 [L35Q G238S E240K]	fragment (N-2 C-7) of SHV-like [Y7W I8V R9I L10F C11P L35Q G238S E240K]	
	
90403945	SHV-12 [L35Q G238S E240K]	fragment (N-9 C-1) of SHV-like [L35Q G238S E240K E288N H289L W290G Q291T]	
	
90403951	SHV-12 [L35Q G238S E240K]	fragment (N-10 C+1) of SHV-like [L35Q G238S E240K A284R A285P L286Y I287K E288N H289L W290E Q291P R292K]	

15718691	SHV-16 [-162.1D -162.2R -162.3W -162.4E -162.5T]	SHV like [H96T Y97H -162.1D -162.2R -162.3W -162.4E -162.5T]	different mutation profile

255045865	SHV-28 [Y7F]	fragment (N-6 C-20) of SHV-1	a fragment with different mutation profile

30230495	SHV-48 [V119I]	SHV-like [L35Q R191H G238S E240K]	different mutation profile
	
33943602	SHV-56 [L35Q K234R]	SHV-like [S14Y L35Q G238S E240K]	
	
83596180	SHV-71[H112Y A146V]	SHV-like [L35Q G238S E240K N254D]	
	
84380855	SHV-86 [L35Q G238S E240R]	SHV-like [Y7F L35Q G238S E240R]	

159138975	SHV-102 [G238A]	fragment (N-24 C-5) of SHV-like [L35Q E168A G251S]	a fragment with different mutation profile
	
159138973	SHV-102 [G238A]	fragment (N-23 C-5) of SHV-like [L35Q E168A G251S]	

154269503	SHV-103 [L250R]	SHV-like [K256R]	different mutation profile
	
161367444	SHV-104 [M5L R202S]	SHV-like [R202S]	
	
257359515	SHV-121 [I8A A22V L35Q M129V]	SHV-like [T18A A22V L35Q M129V]	

256862196	SHV-123 [L35Q G238S E240K P252G N254I]	fragment (C-15) of SHV-like [L35Q G238S E240K P252G N255I]	a fragment with different mutation profile

* SHV-like: SHV variant with a mutation profile that is not in the SHV mutation table

## Utility

A multisequence alignment of all 200 protein entries was generated using CLUSTALW. For protein structures, all sequence entries were included and displayed with aligned secondary structure information. Proteins were labeled by the GIs and linked to the NCBI protein database. Annotation of individual residues is visualized by color-coding in the alignment and upon moving the cursor over the respective residue. The SHVED is accessible at http://www.LacED.uni-stuttgart.de/classA/SHVED by a JavaScript-enabled WWW browser. Protein tables provide information on the protein name, mutation, number of residues missing at the N- and C-terminal (in case of fragments), and on the source organism. As an alternative to the multisequence alignment, the SHV variants are visualized as mutations relative to the sequence of SHV-1. Substitution positions are colored and annotated by the exchanged amino acids.

## Discussion

### Data content of the SHVED

By systematic analysis of protein sequences in the SHVED, 41 protein sequences with a new mutation profile were identified. 22 of them are full length sequences originating from microbial sources and therefore are candidates for a new SHV number assignment. The new mutations occurred either at new position on the sequence or they were new amino acid exchange at already described positions.

### Detection of novel SHV β-lactamases and novel amino acid substitutions

Except for one new mutation profile originating from a synthetic construct (GI 151861), all new mutation profiles originated from microbial sources. As a plasmid-bound gene, the SHV β-lactamase encoding bla_SHV _genes are easily transferred among the members of Gram-negative bacteria, especially Enterobacteriaceae because of their close genetic relationship [6]. Thus, most of the newly detected SHV β-lactamases are from Enterobacteriaceae such as *Klebsiella pneumoniae *(14 SHVs), *Escherichia coli *(15 SHVs), *Enterobacter cloacae *(1 SHV), from both *K.pneumoniae *and *E.coli *(1 SHV), and from both *K.pneumoniae *and *E.cloacae *(1 SHV). Additionally, 3 new SHV variants were found in *Acinetobacter baumannii *and 1 new SHV variant was found in *Salmonella enterica*. Although 19 fragments harbor a new mutation profile, they can not be assigned to a new SHV number because of missing sequence information. However, the information about the substitution at new positions found in these fragments could be used in the future to predict the occurrence of new SHV variants.

### Data inconsistencies and reconciliation

In all 27 cases of inconsistency, the annotated name differed from the actual mutation profile. However, the reasons of the inconsistency varied. In the case of the protein sequence with GI 154269503, the lysine at position 256 is substituted by an arginine, while it is reported that the lysine is exchanged by an arginine at position 250 (K250R) [21]. In the SHV mutation table, it is listed as SHV-103 and characterized by the substitution of a leucine at position 250 by an arginine (L250R). A mutation at position 256 is not yet recorded in the SHV mutation table, and the mutation at position 250 can only be seen in the SHV-103. Probably, the difference in amino acid numbering by the author of GI 154269503 and by the curators of the SHV mutation table at Lahey Clinic caused the inconsistence. In the case of the protein sequence with GI 161367444, the inconsistency might derive from the primer used. In the sequence, only one mutation R202S was found, while it is annotated as SHV-104 which has two mutations (M5L and R202S) according to the SHV mutation table. It is noted in the NCBI entry that the forward primer "ATGCGTTATATTCGCCTGTGTATT" was used to amplify the target DNA, which results a methionine at position 5. Therefore, the deduced amino acid substitution M5L (if it actually occurred) could not be present in the deposited amino acid sequence, and the deposited amino acid sequence should not be annotated as SHV-104 because it does not harbor the mutation profile 'M5L R202S'. In the case of the protein sequence with GI 15718691, the duplication of a pentapeptide 163DRWET167 was reported [22] and assigned as SHV-16. But in addition, two mutations H96T and Y97H are present in the amino acid sequence. Therefore, it is not clear whether the actual SHV-16 harbors only the pentapeptide duplication or additionally the mutations H96T and Y97H. In other cases of inconsistency, the amino acid sequences were submitted to the NCBI protein database without corresponding publication and showed inconsistencies in their annotation. One example is the protein sequence with GI 30230495. It is annotated as SHV-48 which should harbor mutation V119I according to the SHV mutation table, while actually four mutations (L35Q, R191H, G238S, and E240K) were found in the deposited amino acid sequence. In the SHV mutation table, an inconsistency in residue numbering (position 253 and 255) was revealed and communicated to the curator for correction.

## Conclusion

The SHV Lactamase Engineering Database (SHVED) was established to identify new SHV β-lactamases and to identify inconsistencies in public databases. Based on our analysis, 22 candidates for assignment of new SHV names were identified. 27 proteins entries with inconsistencies were found and reconciled. Also, three assigned mutation profiles were identified to be in doubt: SHV-16, SHV-103, and SHV-104. The SHVED thus supports the scientific community to name new SHV β-lactamases and to reconcile existing annotation of SHV β-lactamases sequences.

## Availability and requirements

The SHVED is accessible at http://www.LacED.uni-stuttgart.de/classA/SHVED/ by a JavaScript-enabled WWW browser.

## Abbreviations

GI: GenInfo Identifier; SHVED: SHV Engineering Database

## Authors' contributions

QKT developed the database, built the web pages, analyzed the data, and drafted the manuscript. JP supervised the study and finalized the manuscript. All authors read and approved the final manuscript.

## Supplementary Material

Additional file 1 **Additional_file_1.pdf contains table S1 and table S2 mentioned in the text**. They list new mutation profiles of sequences derived from microbial organisms. Click here for file
