# Supplementary material for: SHV Lactamase Engineering Database: a reconciliation tool for SHV β-lactamases in public databases
Source: BMC Genomics. 2010 Oct 13;11:563. doi: 10.1186/1471-2164-11-563 (PMC3091712; doi:10.1186/1471-2164-11-563)
Supplement: Additional file 1 — Additional_file_1.pdf contains table S1 and table S2 mentioned in the text. They list new mutation profiles of sequences derived from microbial organisms. [file 1471-2164-11-563-S1.PDF]

Table S1: New mutation profiles of full length sequences derived from microbial organisms

Position	6*	7	14*	18	22	35	52*	94*	96*	97*	104*	129	162	166*	191*	202	235	238	240	252	253	256	260	271*	289*
AAREF / GI	R	Y	S	T	A	L	A	K	H	Y	D	M	L	E	R	R	T	G	E	P	N	K	I	S	H
74058441***							T																		
224223446***														K											
154269503																						R**			
160948441																					H**				
223635891***																								I	
161367444																S									
223635893***											G														
223635897		F																S							
259038268																				L**					
41584432																	N**	S	K						
218684515***																									L
51105117	L																	S	K						
30230495						Q									H			S	K						
33943602			Y			Q												S	K						
41584438						Q											N**	S	K						
257359515				A	V	Q						V													
52630976						Q												S	K				V**		
41584428						Q											N**	S	K					N	
224979335						Q		E										S	K						
83596180						Q												S	K		D				
84380855		F				Q												S	R						
15718691									T	H			DRWET insertion												

- *: position is not in the SHV mutation table.
- **: The novel substitution is at known position (in the SHV mutation table).
- ***: Protein sequence contains a new mutation profile in which all amino acid exchanges occur at positions not in the SHV mutation table

Table S2: **New mutation profiles of fragments derived from microbial organisms**

Position	6*	7	8	9*	10	11	35	43	44*	80	89	140	141	168*	213	233*	235	238	240	251*	252	258*	261*	272*	284*	285*	286*	287*	288*	289*	290*	291*	292*
AAREF / GI	R	Y	I	R	L	C	L	R	V	V	E	A	T	E	D	D	T	G	E	G	P	E	V	M	A	A	L	I	E	H	W	Q	R
41584430							Q										N**	S	K														
40950646							Q									E		S	K														
164665324																		S	X														
90403947							Q											S	K										F				
94502905	E	G**	D**	S			Q																							L			
90403949		W**	V	I	F**	P**	Q											S	K														
90403945							Q											S	K										N	L	G	T	
78333												T**	A**																				
90403951							Q											S	K						R	P	Y	K	N	L	E	P	K
157838542								S										S	K														
46309198																					L**												
159138973							Q							A						S													
159138975							Q							A						S													
56463231											Q**							S	K														
56463237										M								S	K														
56463225								S										S	K														
56463229								S			Q**							S	K														
56463235								S							A**		P**	S	K			D	L										
56463239									G	M	Q**							S	K	V				R									

- *: position is not in the SHV mutation table.

- **: The novel substitution is at known position (in the SHV mutation table).
